# The Dentist's Memorandum

**Published:** 1863-01

**Authors:** 


THE DENTIST'S MEMORANDUM: A Book of Engagements and Manual of Ready Reference, for 1863.—BY C. H. CLEAVELAND, M. D.

Those who have seen the former editions of this work need no urging to induce them to buy it. Those who have not should get this forthwith. It is the last of its kind; and an office is not duly "fitted" without it. It is cheap, substantial, and convenient. Address the auther at Cincinnati, O. PRICE $1.00.

